# Semi-quantitative scoring criteria based on multiple staining methods combined with machine learning to evaluate residual nuclei in decellularized matrix

**DOI:** 10.1093/rb/rbae147

**Published:** 2024-12-18

**Authors:** Meng Zhong, Hongwei He, Panxianzhi Ni, Can Huang, Tianxiao Zhang, Weiming Chen, Liming Liu, Changfeng Wang, Xin Jiang, Linyun Pu, Tun Yuan, Jie Liang, Yujiang Fan, Xingdong Zhang

**Affiliations:** National Engineering Research Center for Biomaterials, Sichuan University, Chengdu, Sichuan 610064, China; College of Biomedical Engineering, Sichuan University, Chengdu 610064, China; National Engineering Research Center for Biomaterials, Sichuan University, Chengdu, Sichuan 610064, China; College of Biomedical Engineering, Sichuan University, Chengdu 610064, China; National Engineering Research Center for Biomaterials, Sichuan University, Chengdu, Sichuan 610064, China; College of Biomedical Engineering, Sichuan University, Chengdu 610064, China; National Engineering Research Center for Biomaterials, Sichuan University, Chengdu, Sichuan 610064, China; College of Biomedical Engineering, Sichuan University, Chengdu 610064, China; Neo Modulus (Suzhou) Medical Technology Co., Ltd, Suzhou 215163, China; Neo Modulus (Suzhou) Medical Technology Co., Ltd, Suzhou 215163, China; Kemoshen AI Lab, Shanghai Kemosheng Medical Technology Co., Ltd, Shanghai 201700, China; Kemoshen AI Lab, Shanghai Kemosheng Medical Technology Co., Ltd, Shanghai 201700, China; Sichuan Testing Center for Biomaterials and Medical Devices Co., Ltd, Chengdu 610064, China; Sichuan Testing Center for Biomaterials and Medical Devices Co., Ltd, Chengdu 610064, China; National Engineering Research Center for Biomaterials, Sichuan University, Chengdu, Sichuan 610064, China; College of Biomedical Engineering, Sichuan University, Chengdu 610064, China; Sichuan Testing Center for Biomaterials and Medical Devices Co., Ltd, Chengdu 610064, China; National Engineering Research Center for Biomaterials, Sichuan University, Chengdu, Sichuan 610064, China; College of Biomedical Engineering, Sichuan University, Chengdu 610064, China; Sichuan Testing Center for Biomaterials and Medical Devices Co., Ltd, Chengdu 610064, China; National Engineering Research Center for Biomaterials, Sichuan University, Chengdu, Sichuan 610064, China; College of Biomedical Engineering, Sichuan University, Chengdu 610064, China; National Engineering Research Center for Biomaterials, Sichuan University, Chengdu, Sichuan 610064, China; College of Biomedical Engineering, Sichuan University, Chengdu 610064, China

**Keywords:** decellularized extracellular matrix, nuclear residue, staining method, machine learning, semi-quantitative scoring

## Abstract

The detection of residual nuclei in decellularized extracellular matrix (dECM) biomaterials is critical for ensuring their quality and biocompatibility. However, current evaluation methods have limitations in addressing impurity interference and providing intelligent analysis. In this study, we utilized four staining techniques—hematoxylin-eosin staining, acetocarmine staining, the Feulgen reaction and 4’,6-diamidino-2-phenylindole staining—to detect residual nuclei in dECM biomaterials. Each staining method was quantitatively evaluated across multiple parameters, including area, perimeter and grayscale values, to establish a semi-quantitative scoring system for residual nuclei. These quantitative data were further employed as learning indicators in machine learning models designed to automatically identify residual nuclei. The experimental results demonstrated that no single staining method alone could accurately differentiate between nuclei and impurities. In this study, a semi-quantitative scoring table was developed. With this table, the accuracy of determining whether a single suspicious point is a cell nucleus has reached over 98%. By combining four staining methods, false positives caused by impurity contamination were eliminated. The automatic recognition model trained based on nuclear parameter features reached the optimal index of the model after several iterations of training in 172 epochs. The trained artificial intelligence model achieved a recognition accuracy of over 90% for detecting residual nuclei. The use of multidimensional parameters, integrated with machine learning, significantly improved the accuracy of identifying nuclear residues in dECM slices. This approach provides a more reliable and objective method for evaluating dECM biomaterials, while also increasing detection efficiency.

## Graphical abstract

**Figure:**
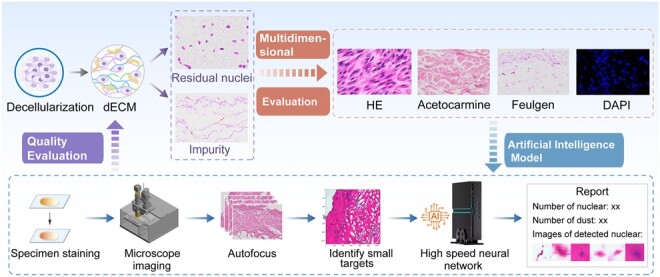

## Introduction

In recent years, with the continuous development of regenerative medicine and tissue engineering, bionic biomaterials have emerged as a prominent trend in biomedical materials research and application. The decellularized extracellular matrix (dECM) biomaterials, due to their ability to retain the biological activity of the original matrix, including its three-dimensional (3D) ultrastructure and specific biomechanical characteristics, influence cellular adhesion, spreading, migration, proliferation and differentiation, making them widely applicable in tissue and organ repair, membrane repair, cell culture scaffolds and bio-inks [1–4]. Currently, although products like dECM are generally classified as medical devices, there are no widely accepted or legally defined standards for quality control and evaluation methods. The clinical purpose of dECM-related products is to replace or repair human tissue while minimizing adverse reactions from allogeneic or xenogeneic biomaterials in the host [5–8]. Therefore, the cell removal rate is a critical indicator of the biocompatibility of dECM products, reflecting the removal of key immunogenic components such as cell membranes, nucleic acids and mitochondria [9–11]. Antigenic epitopes of these residual cellular components are recognized by the host immune system, leading to adverse immune responses or immune-mediated rejection in allogeneic or xenogeneic hosts [12–14]. Studies have shown that residual allogeneic or xenograft cell debris can promote a proinflammatory M1 macrophage phenotype, leading to dense connective tissue deposition or scar formation [15–17]. In addition, damaged mitochondria may release endogenous damage-related molecules (DAMP) that trigger inflammatory responses in the host [18, 19]. However, simply removing immunogenic components from dECM materials is insufficient. Every manipulation of processed tissues or organs inevitably affects the structure and composition of the dECM. While a high cell removal rate in decellularization indicates effective elimination of immunogenic components, it may also compromise key dECM properties, such as the 3D ultrastructure conducive to recellularization and the mechanical strength required for tissue integration [20, 21]. Thus, the cell removal rate is a key index for biocompatibility and quality control, critically impacting the safety and clinical application of dECM. Developing reliable methods to evaluate cell clearance is essential.

Currently, regulatory standards for decellularization quality control have not been systematically established across countries. Crapo suggested establishing a minimum standard of decellularization, including (i) tissue assessment by eosin and hematoxylin-eosin (HE) and 4',6-diamidine-2-phendo (DAPI) staining with no visible nuclei; (ii) any remaining DNA content of no more than 200 base pairs and (iii) the amount of double-stranded DNA should not exceed the dry weight of 50 ng/mg of the material [22]. Two key indicators to evaluate decellularization efficiency are DNA residue and nuclear residue. Residual DNA detection is critical, as DNA is ubiquitous in tissues, can trigger immune responses, and may harbor endogenous retroviruses, making it a reliable quantitative indicator [23–25]. For example, Chinese pharmaceutical industry standard YY/T 1876-2023 [26], Chinese Pharmacopoeia 2020 edition, General Principles 3407, etc provide suitable evaluation methods for DNA residues [27, 28]. Residual nuclei are typically assessed using conventional histological staining or immunofluorescence, serving as a qualitative validation of DNA content. The most common histological staining method used for dECM is HE staining, which colors nuclei blue–purple and extranuclear components red [29, 30]. However, the HE staining method requires a high technical level and testing experience of the testing personnel, and result evaluation remains subjective. Additionally, contamination during preparation, such as from tissue debris, reagents and dust, can significantly affect detection results [31]. Some studies have shown that the use of uncleaned tools or solutions, containers, common solution dye solution, etc will cause contamination [32, 33]. Thus, distinguishing staining contamination from real nuclear residues is essential to avoid false positives and ensure accurate assessment.

Several methods can help to improve the detection of contamination. Although HE staining is commonly used, it is not a specific nuclear staining method. Staining techniques such as acetocarmine, Feulgen and DAPI specifically target nuclei, providing optical signals that differ significantly from HE staining. By comparing these methods, it becomes easier to distinguish nuclear signals from contaminants. Additionally, combining optical data with nuclear features such as position, shape and chroma can further enhance signal reliability. Traditional residual nuclear analysis of dECM relies on pathologists, which is time-consuming and prone to subjective errors, leading to inconsistent results. Machine learning (ML), a branch of artificial intelligence, enables computers to learn from data without explicit programming [34]. In medical diagnostics, AI has demonstrated comparable or superior accuracy to human experts in tasks such as skin and breast cancer detection [35, 36]. Artificial intelligence models based on computer vision and deep learning can achieve highly accurate human–computer interaction by recognizing gestures [37]. ML algorithms are also widely used in image segmentation, recognition and classification [38–41]. For example, a recent study described a deep learning model that enables organ segmentation of head and neck regions from CT images with performance comparable to of experienced radiographers [42].

In this study, HE staining was combined with various nucleophilic dyes, including acetocarmine, Feulgen and DAPI, to evaluate the degree of decellularization, eliminate contamination interference, and quantitatively compare nuclei and contaminated particles in stained sections based on parameters such as area, circumference, longest diameter, shortest diameter, diameter ratio and mean gray scale values. This approach established semi-quantitative scoring criteria for residual nuclei in dECM and addressed the subjectivity and impurity interference associated with HE staining. Additionally, a ML model was trained to intelligently recognize residual nuclei and impurities in dECM. By increasing sample size and sampling from multiple locations, the automatic detection results were optimized, providing insights into future evaluation schemes for intelligent image recognition of residual cell nuclei. This method offers a potential solution for improving quality control and preclinical evaluation of dECM.

## Materials and methods

### Materials setup and preparation

Decellular guide bone regeneration membrane (GBR, Neo Modulus (Suzhou) Medical Technology Co.) was used as the sample group 1. The decellularized pig skin is set to sample 2. Gelatin-polycaprolactone stratified gingival repair membrane [AGM membrane, Neo Modulus (Suzhou) Medical Technology Co.] was cell-free, and was added as a negative control. The raw material of the pig peritoneum with cells of the GBR and the pig skin (No cell removal was performed) were set as positive control 1 and 2. The detailed grouping of samples is shown in Table 1. All the above were fixed in 4% paraformaldehyde for 24 h before dehydration and embedded in paraffin before sectioning. The obtained tissue sections were grouped for HE staining, acetocarmine staining, Feulgen staining, DAPI staining and then scanned using a whole slide scanning system (OLYMPUS, VS 200, Japan).

**Table 1.** Experimental design and grouping

Sample classification	Group	HE	Feulgen	Acetocarmine	DAPI
GBR membrane	Sample 1	15^a^	15	15	15
Decellating pig skin	Sample 2	15	15	15	15
Pig peritoneum	Positive 1	15	15	15	15
Pig skin	Positive 2	15	15	15	15
AGM membrane	Negative	15	15	15	15

a Refers to the number of tissue slices in each group.

### Selection of staining methods for different principles

Tissue sections from each group were stained using HE, acetocarmine, DAPI and Feulgen dyes. These four staining methods complement each other, allowing for accurate identification of nuclei and distinguishing contaminants.

Acetocarmine stains the nucleus red, while dust particles remain black or brown, enabling clear differentiation between nuclei and dust. This method is also simpler than phenol magenta staining [43].

The Feulgen reaction is a classical, DNA-specific staining method that colors the nucleus red or purplish-red, while dust particles retain their natural color [44, 45].

The endogenous fluorescence of nucleic acids is very weak and cannot be used directly studied for nucleic acids using fluorescence technology, but the nucleic acids to DNA by non-covalent bonding. DAPI (4’,6-diamidino-2-phenylindole), a cell-permeable fluorescent probe, is widely used in qualitative and quantitative DNA studies [46, 47]. DAPI is able to insert into the minor groove structure of DNA, especially for binding with DNA in the AT region [48]. When bound to DNA, DAPI’s fluorescence intensity significantly increases, allowing fluorescent nuclei to be easily observed under a fluorescence microscope [49]. In the presence of a nucleus, the DNA will fluoresce blue, making it easy to distinguish from other substances.

### Experimental pollution control

Possible tissue fragment contamination during the staining process is an objective concern. The GBR membrane used in this experiment, composed of decellularized collagen fibers, has a smooth side and a rough side with tentacle-like collagen fibers. This structure increases the likelihood of tissue debris or cell attachment, raising the risk of contamination during the staining process [50, 51]. To prevent contamination from foreign tissue fragments or cells and avoid impacting the research, two pollution control measures were implemented. To prevent exogenous contamination, the solutions for sample fixation, paraffin embedding, and dewaxing were regularly replaced. Containers, embedding boxes, and water tanks were cleaned, and the microtome blade was replaced as needed, with the entire process being monitored onsite. To avoid cross-contamination, sections were cut in this order: negative control, experimental samples, and positive controls. Dewaxing and staining steps were carried out sequentially. If simultaneous processing was required, separate containers were used.

### Multidimensional cell nuclear identification

To clarify the characteristic parameters of the nucleus and distinguish it from non-nuclear substances, this study evaluated its size, shape and color based on six parameters: area, perimeter, longest diameter (Feret), shortest diameter (Minferet), aspect ratio (Feret ratio) and average grayscale value. Stratified sampling was used to randomly select 5 out of 15 positive tissue sections from each staining group, with 5 nuclei and 5 impurities selected per section. Nuclei were selected based on complete shape and uniform color, while impurities were chosen to exclude particles that were too large or too small. The area, perimeter, Feret, Minferet, Feret ratio and average grayscale value of nuclei and impurities were calculated using ImageJ software. The Feret represents the longest inner diameter through the center of the image, the Minferet represents the shortest, and the average grayscale value is the sum of all pixel intensities divided by the total number of pixels. The calculation formula is as follows:


$$\mathrm{Average}\;\mathrm{grayscale}\;\mathrm{value}=\frac{1}{N}\sum_{i=1}^{N}I\left(i\right)$$

In the formula, *I*(*i*) is the grayscale value of the *i*-th pixel in the graph, and *N* is the total number of pixels in the graph.

### Semi-quantitative scoring system for cell nuclei and impurities

Currently, no research exists on a semi-quantitative scoring system for dECM slices. This study innovatively designed a two-part scoring system: one part assesses whether a single suspect point is the cell nucleus, and the other evaluates the overall decellularization effect of the entire section. For a single suspect point, based on specific nuclei, namely positive 1 and positive 2 groups of two nuclei, referred to as nuclear 1 and nuclear 2, with its measured size (area and perimeter), shape (Feret, Minferet and Feret ratio), color (average grayscale value) as a benchmark, in the form of mean ± standard deviation (SD) divided into different score interval. The score was divided into three categories: size, shape and color, with weight factors assigned to each based on their importance. The internal parameter score of each part is the average, and the score of each part is multiplied by the weighting factor and added to the total score, so as to obtain the scoring criterion 1. For each staining method, 25 samples were taken from nuclear 1, nuclear 2 and dust, totaling 300 samples.

The overall decellularization effect of the entire section was evaluated based on three factors: the total number of suspicious points, the number of points identified as nuclei using scoring criterion 1 and the number of suspicious points in the extracellular matrix. The number of suspicious points for each of these three aspects was counted, and the scoring table was divided into different score intervals. Similarly, different proportions were given according to the importance of each part, and then each stained section was scored, and the three parts were added up to get the final score. The sample consisted of 100 slices, with 5 slices taken from each of 5 groups across the staining methods.

### Exploration of automated detection of cell nuclei and impurities based on machine learning

To apply AI techniques for detecting residual nuclei and impurities in dECM sections, we utilized Neuro-Knowledge gene (NK-DNA) multimodal cognitive AI technology, which integrates AI perception algorithms, the Neuro-Knowledge gene engine and other core modules. A deep neural network model was built, and the open AI microscope scanning platform (Kemoshen, RoboScope2301, China) was used for scanning residual nuclei and impurities. The hardware configuration mainly includes an open, expandable intelligent microscope, an automated slide loader, and a high-throughput scanning platform. The open, expandable intelligent microscope features modular hardware and open-source software with AI integration support. It provides engineering examples, supports Python programming, and offers SDK and API documentation. With a field number of up to 26.5, it supports multichannel epi-fluorescence and full motorized operation (resolution: less than 1 micron in the *XY* direction and less than 0.1 micron in focus). It allows automatic full-slide scanning (25 mm × 25 mm), has a 6 mm Z-stage with an optical encoder, a maximum focusing precision of 50 nm, and a universal controller—the controller code is open source and supports upgrades. The slide automation loader (120–300 slides) is programmatically controlled, offering an open SDK and program examples, and supports Python development. The high-throughput scanning system consists of a combination of two devices. A feature dataset was formed by manually labeling the nuclei and impurities in the sample to train the model, and large number of microscopic image datasets are used to train the deep learning model and extract the features. Using a mature AI annotation, training and inference framework, the AI model was optimized for tasks such as image denoising, super-resolution reconstruction, automatic segmentation and analysis, enabling automatic identification of suspicious objects in the sample. In this study, 15 positive samples of Feulgen, HE and acetocarmine slices were used. After manually labeling positive and impurity features, the data were divided into three sets: training, validation and testing, in a 6:2:2 ratio using uniform random sampling with no overlap. The original size 2560 pixels × 2560 pixels image is cut into several segmented images of 1024 pixels × 1024 pixels as the effective dataset for training.

### Statistical analysis

Quantitative data obtained from the experiment were expressed as mean ± SD, with statistical analysis using analysis of variance and comparison between groups using the Tukey test (Tukey *post hoc* tests). All analyses were performed using GraphPad Prism (GraphPad Software, La Jolla, CA). *P* values < 0.05 were considered statistically significant.

## Results

### Residual results of cell nucleus

**Figure 1.**
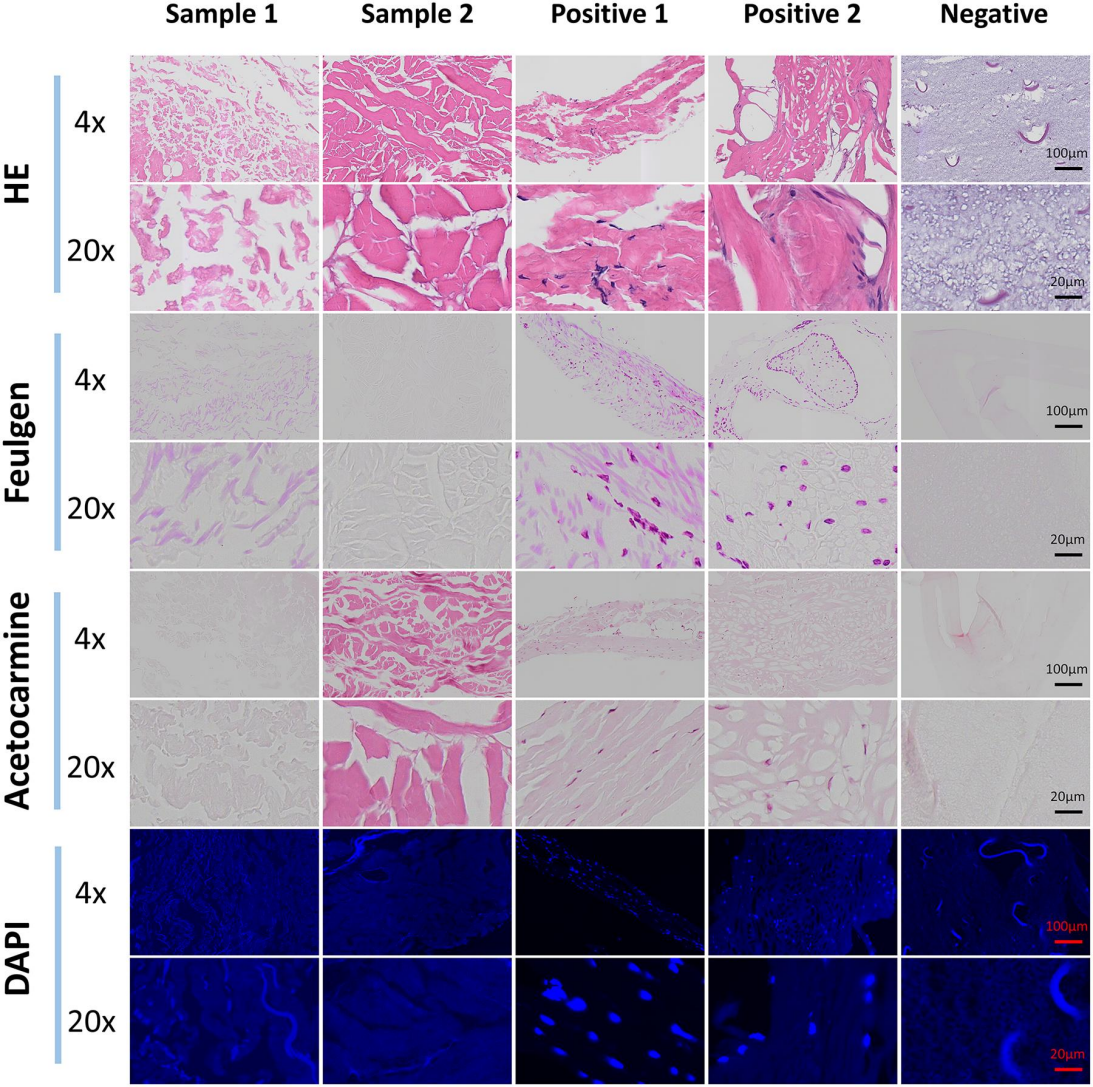
Nuclear residue detection results of four staining methods.

The results of nuclear residue detection of each staining method are shown in Figure 1. In the HE-stained sections, two positive control groups display numerous blue–purple nuclei distributed within a red extracellular matrix, and the negative control group is multiple dyed dark purple small fiber (speculated polycaprolactone component in AGM membrane), but not see the existence of nuclei, indicating that HE staining has certain nuclear residue detection ability.

The Feulgen reaction yielded similar detection results to HE staining. The absence of nuclei in the negative control and the presence of purple–red stained nuclei in both positive controls confirm the Feulgen reaction’s effectiveness in detecting nuclear residues.

Acetocarmine staining revealed numerous red spindled or curved nuclei in the two raw materials of guide bone regeneration membrane, which is in line with the nuclear characteristics of fiber cells in the raw material. In the sample and negative control groups, only lightly stained extracellular matrix components were observed. The above results are similar to the staining results of the previous two methods, indicating that the acetocarmine staining method is also effective.

Unlike the other acid-base dyes, DAPI fluorescence staining indirectly detects DNA via fluorescence technology. The results showed that the two positive controls exhibited strong blue fluorescence from complete cell nuclei. In the negative control and sample groups, blue fluorescence was also present but at a lower intensity than in the positive groups, and no nuclei were detected. These findings suggest that DAPI fluorescence staining is effective in detecting nuclear residues.

### Impurity detection results

Although strict experimental pollution control procedures can greatly reduce the impact of exogenous tissue debris pollution and cross contamination between samples on the detection results of nuclear residues in dECM biomaterials, it remains difficult to completely avoid contamination by particles such as dust, as sample preparation (including embedding, staining and other operations) is conducted in a typical laboratory environment. To assess the impact of contamination on the four staining methods and evaluate their ability to distinguish impurities from real nuclei, suspected dust particles visible to the naked eye in each staining group were compared at 10× and 20× magnifications.

**Figure 2.**
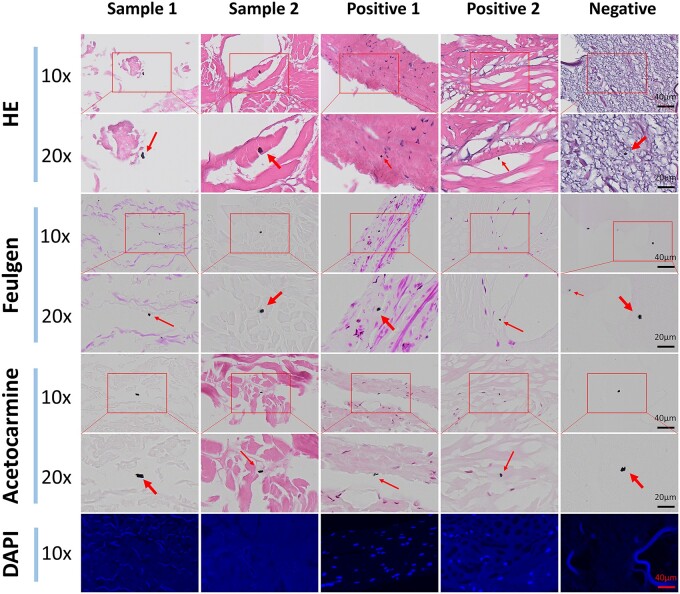
Impurity detection results of four staining methods.

Figure 2 displays suspected dust particles in each group. In the HE-stained group, similar dust particles were present across all sections, highlighting the objectivity of dust contamination in HE staining. Most suspected dust particles were ∼5 μm in diameter and appeared blue–black with irregular staining patterns. In both positive control groups, these particles closely resembled normal nuclei in color and shape, making them difficult to distinguish.

In the Feulgen reaction group, suspected dust particles were also observed in all experimental sections, appearing black or gray with irregular shapes. The suspected dust particles were randomly distributed in the extracellular matrix and its gaps, consistent with the randomness of contamination. In both positive controls, the purple-stained nuclei were clearly distinguishable from the black–gray dust particles, indicating that the Feulgen reaction effectively distinguishes impurities from real nuclei.

Dust-like particles were also present in acetocarmine-stained slices, appearing black and gray like those in the Feulgen reaction, but easily distinguishable from the lighter extracellular matrix and red-stained nuclei. This suggests that acetocarmine staining can also effectively differentiate nuclei from impurities.

No dust particles were observed in DAPI-stained sections, possibly because DAPI binds specifically to DNA, particularly in AT-rich regions, and does not bind to dust particles. Dust particles lack binding sites for DAPI, making them invisible under a fluorescence microscope. Thus, DAPI fluorescence staining can effectively distinguish cell nuclei from impurities.

### Multidimensional parameter identification of cell nucleus and impurities

**Figure 3.**
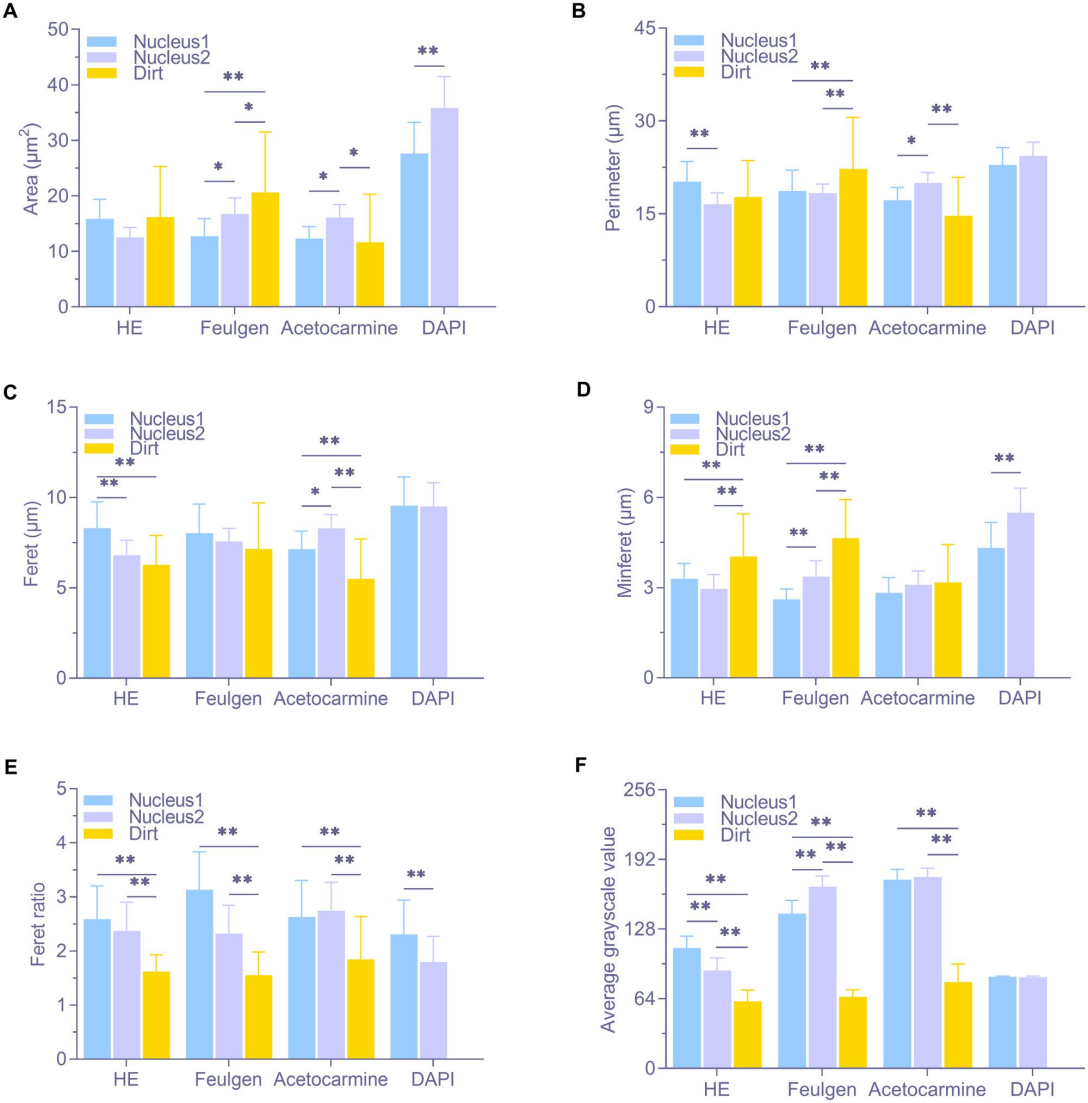
Relevant parameters for nuclei and impurities in the group positive for the four staining methods. (**A**) Area. (**B**) Perimeter. (**C**) Feret. (**D**) Minferet. (**E**) Feret ratio. (**F**) Average grayscale value (* *P* < 0.05, ** *P* < 0.01, *n* = 25).

Figure 3A and B presents a comparison of the size parameters, including area and perimeter, of cell nuclei and contaminating particles on two types of bone-guided regeneration membranes. Figure 3A illustrates the differences in area size among the three staining subjects (nucleus 1, nucleus 2 and dust) across each staining method. No statistical differences were observed among subjects in the HE group, but there were no differences between the two nuclei in the other three staining groups. Except for nucleus 1 in the acetocarmine group, the nuclei and impurities differed between the Feulgen and acetocarmine groups. Additionally, the area of the same staining object was similar across the three staining groups, while the nucleus area in the DAPI group was significantly larger than in the other groups. Figure 3B shows the differences in circumference among the staining subjects. Differences were only observed between the two nuclei in the HE group. No differences were observed between the nuclei in the Feulgen group, but significant differences were found between nuclei and impurities. In the acetocarmine group, differences were observed between nucleus 2 and nucleus 1 or impurities, but no differences were found between nucleus 1 and impurities. The circumference of the nucleus in the DAPI group remained larger than that in the other three groups.

Figure 3C–E shows the size of the nuclei and the dust shape parameters, including Feret, Minferet, and Feret ratio. Figure 3C shows the difference in the longest diameter of the stained objects. The HE group showed significant differences between nucleus 1 and the other two stained objects, but no difference between nucleus 2 and contaminating particles. There were no differences within the feulgen and DAPI groups, but there were significant differences within the acetocarmine group. The Feret of the nucleus in the DAPI group was greater than that in the other groups. Furthermore, the Feret of impurities in each staining group was smaller than the nucleus in each method group. Figure 3D shows the difference in the Minferet of the stained objects. There was no difference within the acetocarmine group, while both nuclei were significantly different from the impurities in the HE and Feulgen groups. There were also significant differences between the two cell nuclei in the Feulgen and DAPI groups. In addition, the Minferet of nucleus was still the largest in the DAPI group. The shortest diameter of the impurities in each group was greater than the nucleus. Figure 3E shows the difference in the Feret ratio (longest/shortest diameter). Overall, except for the DAPI group, the Feret ratio of two types of cell nuclei in the three staining method groups is greater than 2, while that of the impurities is closer to 1, and there is a significant difference between the two. This indicates that the two nuclei are more striped, spindle and curved, while the impurities are rounder. The nuclear Feret ratio in the DAPI group is smaller than the other three groups and tends to be more spherical.

Average grayscale value is often used in image processing to describe the average degree of gray scale (brightness) in a certain area of the image. By converting color images into grayscale images, multi-parameter images such as RGB can be transformed into single-color parameter maps to facilitate mutual comparison. The gray scale values usually range from 0 (black) to 255 (white). Figure 3F shows the size of the color parameters of the impurity and nuclei, expressed as mean gray order values. Except for acetocarmine and DAPI groups, the other two groups. There were significant differences between nuclei and impurities in each group, and the mean gray scale values of both nuclei were much larger than that of impurities. Overall, the average gray scale value of the impurity is more inclined to 0 (black).

### Semi-quantitative scoring system for cell nuclei and impurities

**Figure 4.**
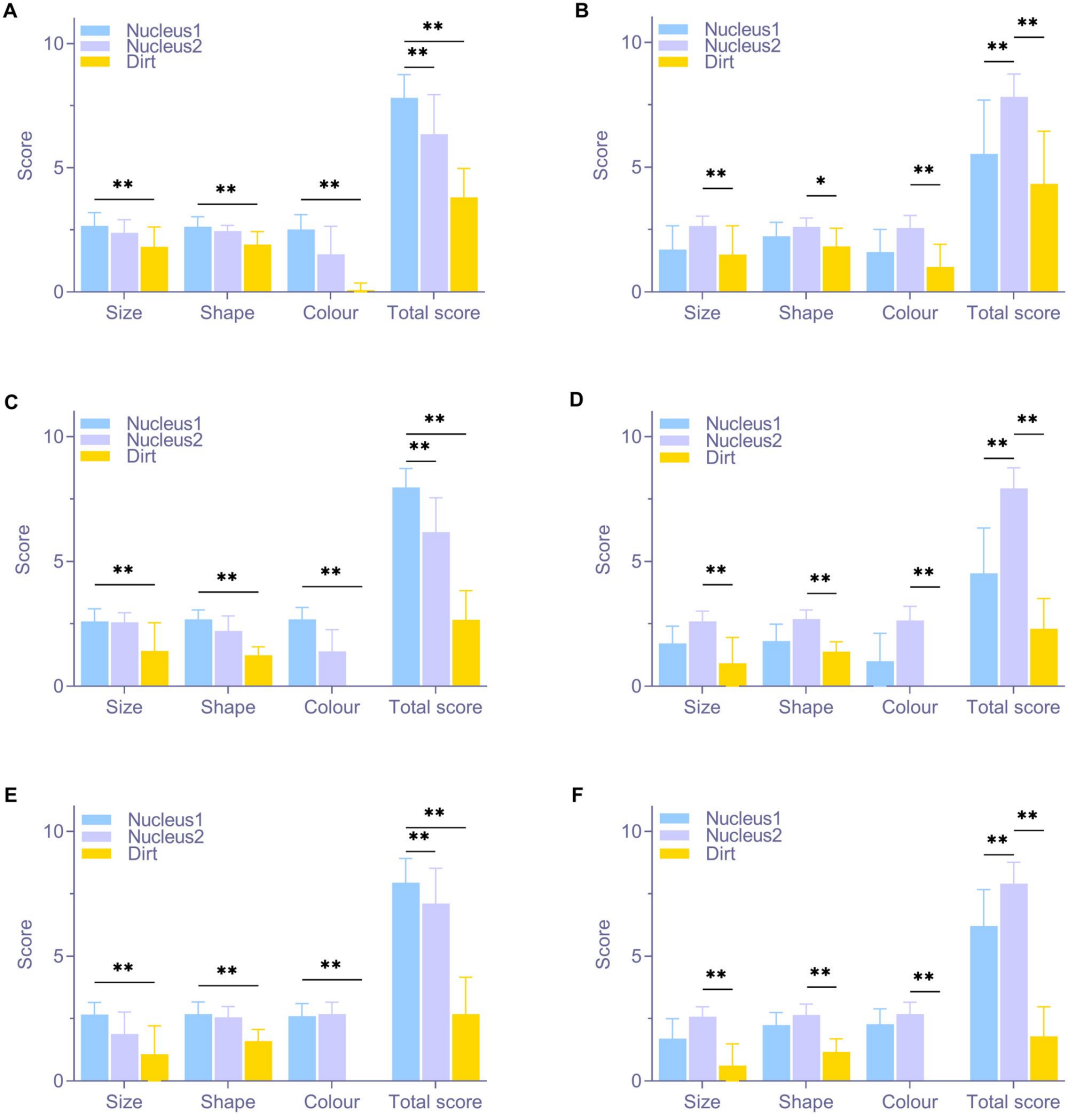
Semi-quantitative scoring results of whether a single suspicious point in the section is a nucleus. (**A**) The result of HE group based on nucleus 1. (**B**) The result of HE group based on nucleus 2. (**C**) The result of feulgen group based on nucleus 1. (**D**) The result of feulgen group based on nucleus 2. (**E**) The result of acetocarmine group based on nucleus 1. (**F**) The result of acetocarmine group based on nucleus 2 (* *P* < 0.05, ** *P* < 0.01, *n* = 25).

Table 2 presents the scoring table for determining whether a single suspicious point is a cell nucleus. In Table 2, different scoring intervals are established based on the characteristic parameters of nuclear 1 and nuclear 2, using mean ± SD. The closer a suspicious point’s parameters are to those of the reference nucleus (i.e. closer to the mean), the more likely it is to be a nucleus, resulting in a higher score. Figure 4 shows the scoring results for identifying whether a single suspicious point in the slice is a cell nucleus, according to the scoring system in Table 2. DAPI fluorescence staining was excluded, as it cannot visualize contaminant particles under the microscope. For the other three staining methods, significant differences in size, shape and color scores were observed between contaminant particles and reference nuclei, regardless of whether nucleus 1 or nucleus 2 was used as the reference. The total score for contaminant particles was significantly lower than that of the reference nucleus, suggesting that the semi-quantitative scoring method in Table 2 effectively identifies whether suspicious points are impurity particles across all three staining methods. However, scores for non-baseline nuclei were lower than those for baseline nuclei, and in some groups, they were close to those of contaminant particles (Figure 4A, B, and D), indicating limited discrimination for non-baseline nuclei.

**Table 2.** Multidimensional semi-quantitative scoring

Scoring object	Evaluation dimensions		Scoring				
			0	1	2	3	4
Individual suspect scoring	Size^a^	Areas	Values are outside 3*σ*^b^	Values are between 2*σ* and 3*σ*	Values are between 1*σ* and 2*σ*	Values are within 1*σ*	
		Perimeter					
	Shape	Feret					
		Minferet					
		Feret ratio					
	Color	Average grayscale value					
The acellular effect of the whole section scoring	Number of overall suspicious spots		0	Rare, 0–10 slices per sheet	Some, 10–20 slices per sheet	Medium, 20–50 slices per sheet	Vast, more than 50 slices per sheet
	Number of suspicious spots that may be nucleus^c^		0	Rare, 0–2 slices per sheet	Some, 2–5 slices per sheet	Medium, 5–10 slices per sheet	Vast, more than 10 slices per sheet
	Number of suspicious spots located in the extracellular matrix		0	Rare, 0–5 slices per sheet	Some, 5–10 slices per sheet	Medium, 10–20 slices per sheet	Vast, more than 20 slices per sheet

a The scores of each dimension are the average of the scores of its characteristic parameters.

b *σ* is SDs; all values are compared to the parameters of the nucleus.

c This component is considered to be more important in the evaluation of nuclear residue, so its score is multiplied by a weight factor of 2.

**Figure 5.**
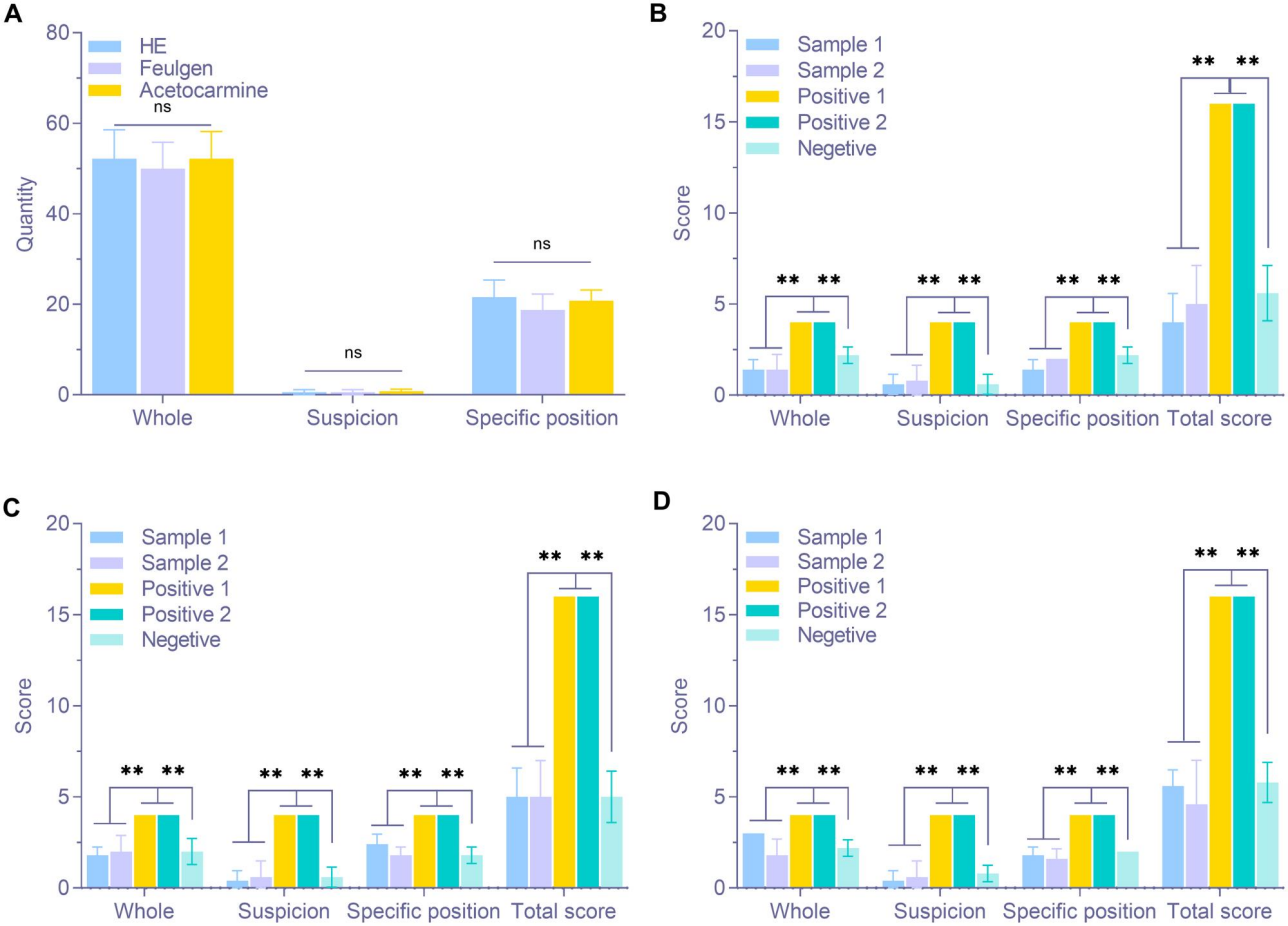
Semi-quantitative scoring results of acellularization effect of the whole section. (**A**) The benchmark. (**B**) The HE group. (**C**) The Feulgen group. (**D**) The acetocarmine group (* *P* < 0.05, ** *P* < 0.01, *n* = 5).

Table 2 also includes the scoring table for evaluating the decellularization effect of the entire slice. Statistical analysis of negative controls in each group (Figure 5A) ensured accurate scoring intervals. A weighting factor of 2 was added to the number of suspicious nuclei identified by scoring criterion 1, as residual nuclei are the most direct indicator of decellularization and hold greater importance than other factors. Figure 5 presents the evaluation results of the decellularization effect for each staining method, based on the scoring system in Table 2. The positive group’s scores in each staining method were significantly higher than those of the negative and sample groups, indicating that the evaluation criteria for decellularization met expectations. No significant differences were observed between the sample and negative groups, confirming that both dECM samples exhibited effective decellularization.

### Exploration of automated detection of cell nuclei and impurities based on machine learning

**Figure 6.**
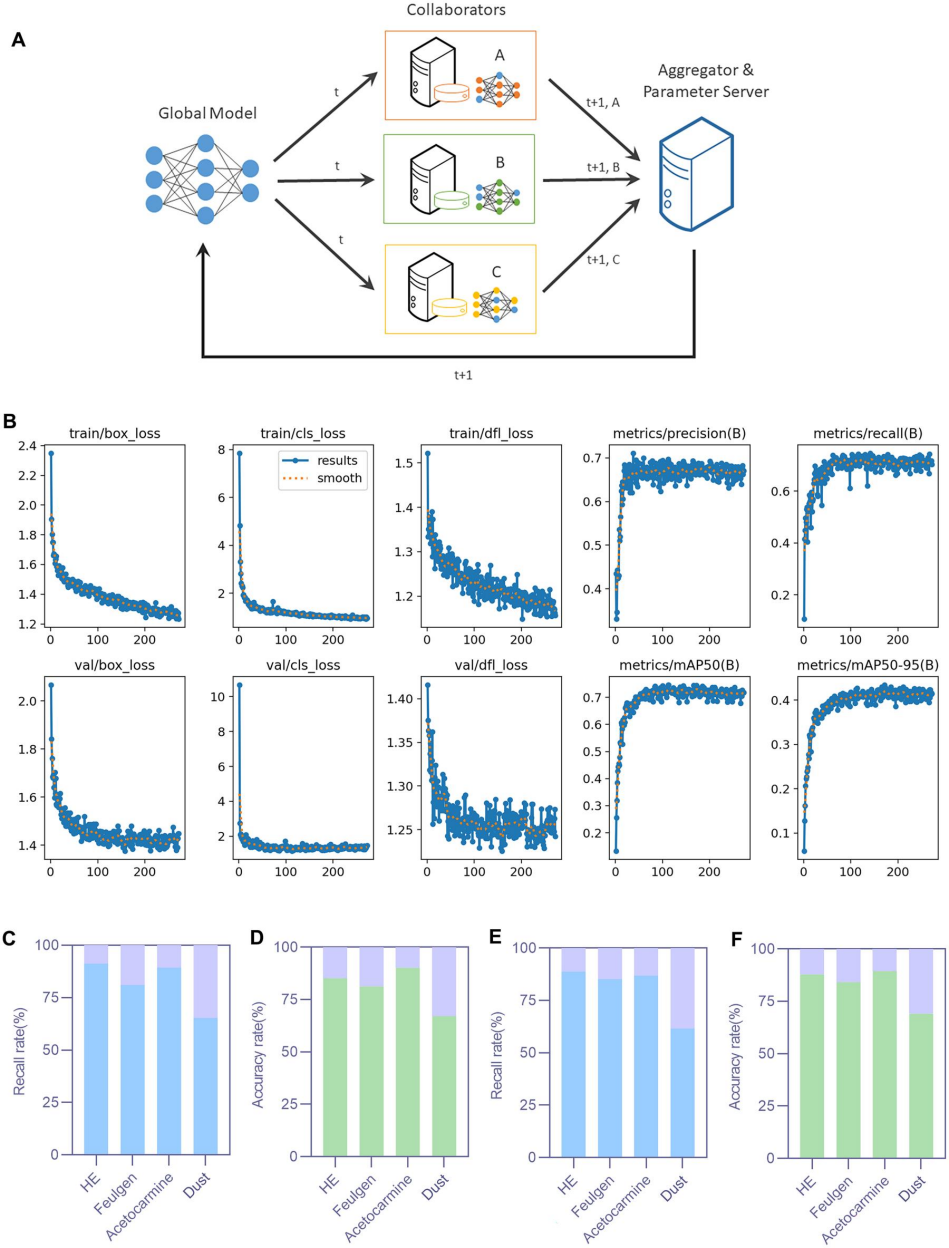
Machine learning results. (**A**) Reasoning and deployment process. (**B**) The model training map. (**C**) The recall rate of model validation. (**D**) The accuracy rate of model validation. (**E**) The recall rate of model testing. (**F**) The accuracy rate of model testing.

Figure 6 shows the ML automatic detection results of residual nucleus and impurity recognition in dECM tissue slices. The link to the code and dataset used in this study can be accessed at https://github.com/Meng228629/letter-code.git. Figure 6A describes the process of distributed ML, which is composed of global model, collaboration service and parameter aggregation server. The more sensitive training data (such as the shape, size and color parameters of nuclei and impurities) are stored locally. The updated model parameters (*t* + 1) are obtained through the collaboration service, and many updated parameters are sent to the aggregation server to form a new parameter set and sent back to the global model for updating, completing a round of training iteration. As can be seen from the training diagram of the model, with the increase of epoch, the model training results gradually stabilize. After 272 epochs, the model optimal index (Figure 6B) is reached at 172 epochs. From the validation data of the model, we can see that the recognition recalls rate and accuracy of HE, Feulgen and Acetocarmine staining methods are high, and the recognition accuracy of dust is low. The model test results show that the recall rate and accuracy of tissue sections of HE, acetocarmine and Feulgen are above 0.8 (Figure 6C–F).

## Discussion

In this study, we combined the traditional HE staining method for nuclear residue detection of dECM materials with three additional nucleophilic staining methods (Feulgen reaction, acetocarmine staining, DAPI fluorescence staining). The experimental design involved staining materials with different properties (AGM membrane, two GBR membranes and their raw materials) to observe and compare the effectiveness of each staining method in detecting nuclear residues, while also examining the impact of impurity contamination. Additionally, we explored the semi-quantitative scoring system and intelligent identification methods for detecting and evaluating nuclear residues and impurities in dECM materials.

Pathological staining processes often exhibit contamination with exogenous tissue fragments [52, 53]. Experimental results showed that, after controlling sectioning and staining sources, no large tissue fragments with multiple cell nuclei were observed in any group. This demonstrates that contamination can be effectively avoided, thereby improving the reliability of nucleophilic staining detection results.

Figure 1 shows that HE staining effectively assesses general decellularization of dECM material, including intact cell structures and nuclear component removal. However, impurity contamination can still significantly affect detection results (Figure 2). Suspicious staining spots appear black or blue–black under the microscope, differing in color from the scanned image (mostly blue). Additionally, some suspicious staining spots appear at a different focal level than the sample under the microscope. When the sample is in focus, suspicious spots appear blurred, with a lighter blue or blue–black color. In contrast, when the spots are in focus, they appear black or black-edged with blue–black centers. Moreover, the spots are opaque with uniform coloring, which does not align with nuclear membrane or internal chromatin characteristics. Thus, these staining spots are identified as dust particles, not nuclei.

Visually, suspected dust particles resemble normal cell nuclei in color, increasing the risk of misidentification as residual nuclei. The prevalence of dust contamination on slices (with dozens of particles per HE-stained section) can lead to false-positive results. The residual status of cell nuclei is a key indicator of decellularization, directly impacting dECM biomaterial safety and quality control. If residual nuclei in dECM are assessed only by HE staining, false positives from impurities could hinder product commercialization.

A staining method that colors nuclei in hues other than blue or blue–purple would aid in distinguishing dust and other contaminants, reducing detection errors. Additionally, staining methods based on different principles should be used to confirm nuclear identification. This study selected three nucleophilic staining methods with different principles: Feulgen reaction, acetocarmine staining and DAPI fluorescence staining.

To evaluate the impact of impurity contamination on the three staining methods and assess each method’s ability to distinguish impurities from real nuclei, suspected dust particles were identified in each experimental group. No visible suspected dust particles were observed in the DAPI groups (Figure 2). Besides its inability to bind impurities, DAPI emits low-intensity fluorescence even before binding to DNA. The low-intensity blue fluorescence emitted by DAPI on the background plate masks the gray–black impurities, making them difficult to observe under fluorescence microscopy. Thus, impurities had minimal impact on DAPI fluorescence staining. In the other two staining methods, suspected dust particles and cell nuclei differed significantly in color, shape and distribution (Figure 2). Color-wise, suspected dust particles appeared black or blue–black, similar to those in HE staining, while nuclear components in the guide bone regeneration membrane stained red or purplish–red, providing a sharp contrast. Suspected dust particles had irregular shapes, including circles and bars, while most cell nuclei in the raw material were striped, fusiform or curved, consistent with the typical shape of fiber cell nuclei. The distribution of suspected dust particles in the extracellular matrix was irregular: some were within the matrix, some adjacent to it, and others appeared suspended above the matrix plane, making them difficult to focus under the microscope. In contrast, nuclei were typically embedded in the extracellular matrix and aligned at the same focal level. These results suggest that dust contamination had less impact on Feulgen reaction, acetocarmine staining and DAPI fluorescence staining compared to HE staining. These three affinities staining methods effectively distinguish nuclei from impurities, minimizing the impact on dECM detection results.

The qualitative analysis above indicates that nuclei differ from impurities in color, shape and distribution. To further clarify these differences, a quantitative statistical comparison of size (area and perimeter), shape (Feret, Minferet, Feret ratio) and color (average grayscale value) was conducted on four nucleophilic-stained sections and suspected dust particles, exploring a multi-parameter approach for nuclear identification. In terms of size, the nuclei of the three staining methods (except DAPI) were relatively similar (Figure 3). The area and perimeter of specific nuclei in these methods allowed for the differentiation of different cell types. Additionally, the standard deviations of area and perimeter for suspected dust particles were larger than those of the nuclei (Figure 3), indicating greater size variability among impurities, consistent with the random nature of contamination. Notably, only in the HE group were the area and perimeter of the nuclei statistically similar to those of the suspected dust particles, confirming that impurity sizes in HE staining can resemble those of the nuclei.

Regarding overall shape, nuclei in the three groups (excluding DAPI) had larger Feret and smaller Minferet values compared to suspected dust particles, indicating shape differences (Figure 3). The Feret ratio indicated that nuclei were more strip-like, spindle-shaped or curved, while impurities were more rounded. Moreover, except in the acetocarmine group, the Feret ratio differed between the two types of nuclei, indicating this parameter’s ability to distinguish them. In terms of color, the average grayscale value of impurities was lower, appearing darker, while nuclei had higher values, showing a significant difference between the two. This suggests that average grayscale value can effectively differentiate nuclei from impurities. Additionally, in the HE and Feulgen groups, the average grayscale values of different nuclei differed significantly, unlike in the acetocarmine and DAPI groups. This suggests that HE staining and the Feulgen reaction can better differentiate chromatin based on color, likely due to varying affinities of tissue components for dyes and different staining properties [54, 55]. Nuclei in the DAPI group differed significantly from those in the other three staining methods in terms of size, shape and color, likely due to the characteristics of fluorescent dyes. In summary, area and perimeter parameters can differentiate the sizes of nuclei and impurities, the Feret ratio reflects the general shape of the stained object, and Feret and Minferet parameters provide additional shape differentiation. The average grayscale value indicates color differences, clearly distinguishing nuclei from suspected dust particles.

Using this multi-parameter method for nuclear identification, this study developed a semi-quantitative scoring system for residual nuclei based on nuclei from two positive samples. Verification of the original sections demonstrated that the semi-quantitative scoring method in Table 2 is effective for single suspicious points, accurately distinguishing impurities from nuclei. While the scoring system is effective, identifying impurities in multicellular dECM materials may be challenging, as non-benchmark cells could be misjudged as impurities. This issue can be resolved by incorporating additional reference nuclear types. The scoring results for the overall decellularization effect of both positive and negative samples met expectations, and increasing the number of recognition targets reduced overall error. Both Feulgen and acetocarmine staining methods, which are easily identifiable by the human eye, as well as HE staining, which is more affected by impurities, effectively identified decellularization, confirming the scoring system’s effectiveness for large-area tissue sections. The accuracy of the semi-quantitative scoring system can be enhanced by adding more nuclear types as benchmarks and refining the weighting of each scoring component based on practical needs.

Like the HE staining method, some limitations of the other three staining methods were also found. In the group 1 positive for Feulgen reaction in this experiment (Figure 1), the extracellular matrix part was colored light pink, presumably because its extracellular matrix contained of aldehyde, aldehyde, ketols, alcohol and acetal phospholipid that could react with Schiff reagent [56]. In addition, the influence of acid hydrolysis time and temperature on nuclear staining may interfere with the detection of nuclear residues when the nuclear cytoplasmic color is similar [57, 58]. Furthermore, differences in the alkaline magenta components used to prepare the Schiff reagents and contamination with the dye reagents may also affect the assessment of Feulgen staining [59]. The extracellular matrix components stained by acetocarmine staining are lighter (Figure 1). If the nuclear status of dECM material is detected by magenta acetate stain alone, it may not be possible to accurately locate the section tissue due to shallow matrix staining. In this experiment, a large area of blue fluorescence appeared on some DAPI stained sections, which may be caused by the unwashed DAPI remaining on the background plate. DAPI, as a fluorescent dye, also has the problem of fluorescence quenching [60]. If it cannot be detected as soon as possible after staining, it may cause fluorescence quenching, resulting in a weakened fluorescence signal and affecting the image quality. In addition, because DAPI is more sensitive to the detection of DNA, if microbial contamination occurs, there may be blue fluorescent spots of 1 μm or less, which will affect the result judgment.

In addition to commonly used chemical staining methods, techniques based on molecular detection, such as DNA fragment length analysis, intracellular molecular quantification, and DNA quantification analysis (e.g. spectrophotometry and fluorescence-based assays), as well as *in vivo* implantation experiments, can also be utilized for evaluating residual cell nuclei in dECM [47, 61, 62]. These molecular detection-based methods provide precise measurements of residual DNA and detect small fragments to ensure the removal of larger immunogenic fragments, offering high sensitivity for decellularization assessment [63, 64]. In this study, we utilized a combination of multiple staining methods, each targeting different cellular structures, to accurately differentiate nuclei from impurities. HE and acetocarmine staining visualize nuclear structures and distinguish them from extracellular components, while Feulgen reaction and DAPI staining provide specificity for DNA content, eliminating false positives caused by impurities. Additionally, staining methods has a low cost and only requires inexpensive reagents and basic equipment. It can also serve as a universal standard, combined with molecular detection technology to provide more accurate data for clinically relevant products.

The approach demonstrated in this study not only enhances the existing evaluation criteria for residual nuclei in dECM but also offers a scientifically sound and practical method that can complement molecular detection techniques. By integrating these staining methods with ML, this strategy contributes to improving the accuracy and efficiency of residual nuclei detection, supporting quality control efforts for dECM-related products.

With the development of ML techniques, automated identification of nuclei became possible. Suspected dust particles and nuclei were quite different in color, shape and distribution. This information can be extracted by applying image processing and computer vision techniques as nuclear features, thus training the AI to determine whether there are residual nuclei on the slice. By using a variety of staining methods and increase the range and number of detection section, ML is expected to solve the detection of subjectivity, time consuming and ambiguous results, greatly improve the dECM biomaterial residual cell nucleus detection efficiency and accuracy.

In this study, the relevant parameters of the size, shape and color of nuclei and impurity particles in different staining methods were obtained by quantitative analysis, which can be used as relevant learning indicators of ML, learning to extract features trained to judge the decellularized matrix biofilm material without nuclear residue and impurities of ML model. In this study, the constructed model initially proved the ability to detect suspicious objects by training a small sample size dataset. Moreover, the detection efficiency has been greatly improved. The complete detection cycle took 4–5 min per slide, and the recall rate of cell nucleus identification reached 82.6%, and the accuracy rate reached 88.1%. The small data size is one of the important reasons for the effect of model identification. The low coverage of target instance features is low, which affects the recognition ability and generalization ability of the model. Expanding the scale of the annotated data to about 100 glass slide samples, it is expected that the identification recall rate/accuracy will reach more than 95%. In addition, the accuracy of the slice sampling range and the quality of manual data annotation in the early stage will also affect the accuracy of the model. The missing labeling in the process of data sample annotation and the mislabeling will affect the correctness of the dataset, which is obvious in the data annotation of Feulgen staining, leading to the low accuracy of model identification. In addition, the 3.2 million parameter model adopted in the model verification stage will adopt 25.9 million parameter models later, and the model will be adjusted at the same time, which can improve the example identification ability while taking into account the processing efficiency. In fact, we believe that improving identification efficiency may be more important in later research and industrial applications. Because, if it is possible to effectively establish the link between the multi-sections cell residue of the raw material/product and its clinical application effect/safety, it is possible to provide strong support for further upgrading of such products.

Unlike other commonly used ML algorithms, our unique Neuro-Knowledge Gene (NK-DNA) multimodal cognitive AI technology integrates relevant AI perception and cognition algorithms. It offers open access to its capabilities and resources through API/SDK, providing cloud and integrated multi-interface support. This technology enables applications with cognitive and real-time interactive learning capabilities. Combined with an open high-precision microscopic scanning platform and a mature AI annotation, training, and inference framework, it performs exceptionally well in tasks such as image denoising, super-resolution reconstruction, automatic segmentation and analysis. In comparing our ML model for nucleus and impurity detection with other commonly used models, we evaluated Support Vector Machine (SVM), Random Forest (RF), Convolutional Neural Network (CNN) and U-Net on identical datasets. Each model showed unique strengths, yet our approach demonstrated superior accuracy and specificity, particularly in distinguishing between nuclei and impurities. SVM and RF provided robust results for structured data but struggled with the high dimensionality of multi-staining images, resulting in a lower specificity. CNN performed well in shape and boundary recognition, though it lacked the nuanced feature extraction required for subtle impurity differentiation. U-Net, while effective for nucleus segmentation, had limitations in correctly identifying ambiguous impurities that mimic nuclear morphology. Our model, integrating open high-precision microscopic scanning and mature AI annotation, training, and inference frameworks, achieved recognition accuracy of over 90%, outperforming other models by effectively reducing false positives. These results highlight our model’s robustness in handling complex staining data, making it a promising tool for accurate and efficient residual nucleus detection in decellularized matrix evaluations.

This study aimed to address the core research question (RQ) of whether a multidimensional scoring system, combined with ML, could effectively improve the accuracy and reliability of detecting residual nuclei in dECM. Our findings support this approach, demonstrating that the integration of multiple staining methods (HE, Feulgen, acetocarmine and DAPI) provided a significant advantage over single-method staining by reducing false positives caused by impurities. Specifically, the combined staining approach enabled our model to differentiate nuclei from dust particles and other contaminants with an accuracy improvement of over 10% compared to using HE staining alone. Furthermore, the ML model trained on multi-stained image data achieved a recognition accuracy of over 90% for detecting residual nuclei, indicating robust performance in correctly identifying nuclear residues. These results validate the feasibility and effectiveness of our approach for dECM quality assessment, offering a reliable tool that could enhance clinical safety and consistency in dECM applications.

## Conclusion

To improve the reliability of detecting residual nuclei in dECM materials, the comprehensive use of multiple nucleophilic dyes, in addition to HE staining, helps mitigate the limitations of individual methods, while the quantitative identification of nuclei through multi-parameter analysis can effectively reduce the impact of impurity contamination. Additionally, extracting nuclear features using multi-parameter dimensions and applying ML for the intelligent identification of residual nuclei and impurities can enhance the accuracy of detection, reduce dependence on inspector expertise and improve overall detection efficiency.
